# Maackiain Mimics Caloric Restriction through *aak-2*-Mediated Lipid Reduction in *Caenorhabditis elegans*

**DOI:** 10.3390/ijms242417442

**Published:** 2023-12-13

**Authors:** Saveta G. Mladenova, Monika N. Todorova, Martina S. Savova, Milen I. Georgiev, Liliya V. Mihaylova

**Affiliations:** 1 Independent Researcher, 1000 Sofia, Bulgaria; savimladenova@abv.bg; 2 Laboratory of Metabolomics, Institute of Microbiology, Bulgarian Academy of Sciences, 139 Ruski Blvd., 4000 Plovdiv, Bulgaria; mntodorova@yahoo.com (M.N.T.); m.sav@abv.bg (M.S.S.); milengeorgiev@gbg.bg (M.I.G.); 3 Department of Plant Cell Biotechnology, Center of Plant Systems Biology and Biotechnology, 4000 Plovdiv, Bulgaria

**Keywords:** obesity, lipid accumulation, *Caenorhabditis elegans*, orlistat, maackiain, AMPK

## Abstract

Obesity prevalence is becoming a serious global health and economic issue and is a major risk factor for concomitant diseases that worsen the quality and duration of life. Therefore, the urgency of the development of novel therapies is of a particular importance. A previous study of ours revealed that the natural pterocarpan, maackiain (MACK), significantly inhibits adipogenic differentiation in human adipocytes through a peroxisome proliferator-activated receptor gamma (PPARγ)-dependent mechanism. Considering the observed anti-adipogenic potential of MACK, we aimed to further elucidate the molecular mechanisms that drive its biological activity in a *Caenorhabditis elegans* obesity model. Therefore, in the current study, the anti-obesogenic effect of MACK (25, 50, and 100 μM) was compared to orlistat (ORST, 12 μM) as a reference drug. Additionally, the hybrid combination between the ORST (12 μM) and MACK (100 μM) was assessed for suspected synergistic interaction. Mechanistically, the observed anti-obesogenic effect of MACK was mediated through the upregulation of the key metabolic regulators, namely, the nuclear hormone receptor 49 (*nhr-49*) that is a functional homologue of the mammalian PPARs and the AMP-activated protein kinase (aak-2/AMPK) in *C. elegans*. Collectively, our investigation indicates that MACK has the potential to limit lipid accumulation and control obesity that deserves future developments.

## 1. Introduction

Obesity is a complex pandemic-scaled disease, which is a significant factor leading to numerous comorbidities including cardiovascular disease, type 2 diabetes, hypertension, stroke, neurodegenerative disease, metabolic disorders, and certain cancers [1,2,3]. Over 4 million deaths per year are attributed to obesity-derived diseases [3,4,5,6,7]. The development of effective obesity therapy is an enormous challenge due to its complex etiology and pathophysiology [1,5,6,7,8]. Moreover, the side effects of the long-term prescription of approved anti-obesity medicines, such as gastro-intestinal disturbances, as well as insufficient efficacy encourage the development of novel approaches for obesity management [2,9]. As natural products are widely-accepted as safe, the identification of plant-based compounds that regulate energy and lipid metabolism offers a promising perspective [10,11,12,13]. Plant secondary metabolites may help restore dysregulated metabolic signaling pathways in obese patients through various mechanisms. For instance, they can inhibit lipid synthesis and/or promote the elevated degradation of accumulated triglycerides, as well as enhance energy expenditure. Moreover, the modulation of signaling pathways involved in feeding behavior could also benefit impaired metabolic signaling during obesity [2,12,13].

Our previous study, performed in human adipocytes, provided substantial evidence that maackiain (MACK), a natural pterocarpan, strongly inhibits adipogenesis. The observed effect was mediated via the downregulation of the essential for adipocyte differentiation peroxisome proliferator-activated receptor gamma (PPARγ) and CCAAT/enhancer binding protein alpha C/EBPα signaling, along with the decrease in lipogenesis through the inhibition of sterol regulatory element-binding protein 1 (SREBP1) and its downstream target—acetyl-CoA carboxylase [14]. Moreover, several reports suggest that MACK-containing extracts possess anti-diabetic activity [15,16], which highlights the potential of MACK in the therapy of metabolic disturbances. Considering the insufficient number of studies addressing the anti-obesogenic potential of MACK and to further validate our hypothesis in vivo, an investigation was performed in an obesity model of glucose-supplemented *Caenorhabditis elegans* [17,18]. In addition, a hybrid combination between the anti-obesity drug orlistat (ORST) and MACK at the highest experimental concentration was assessed for a putative synergistic interaction.

Decades of research have proven *C. elegans* as a reliable platform for evaluating the bioactivity of both natural and synthetic substances in relation modulation in the neuroendocrine system and energy metabolism [19,20,21]. The functionally conserved genes involved in lipid and carbohydrate metabolism between *C. elegans* and mammals play a crucial role in obesity research [22,23]. The high extent of homology between key human energy regulators such as AMP-activated protein kinase (AMPK), sirtuins (SIRT1), SREBPs, PPARs, and PPAR-gamma coactivator 1 alpha (PGC-1α), and those in *C. elegans*—AAK-1/-2, SIR2.1, SBP-1, NHR-49, and MDT-15, respectively, provides additional rationale for a deep mechanistic investigation of the modulation of lipid metabolism [24,25,26,27]. Furthermore, it has been described that AAK-2 stimulates lipid mobilization in *C. elegans* [7,21,26]. Additionally, SBP-1 orchestrates fatty-acid homeostasis by modulating the expression of lipogenic enzymes, whereas NHR-49 and MDT-15 initiate the expression of downstream target genes involved in fatty-acid β-oxidation and desaturation [25].

The current study aimed to evaluate the lipid-reducing effect of MACK in glucose-supplemented *C. elegans* as an obesity model platform and to enlighten the molecular pathways involved. The evaluation of locomotion, chemotaxis, lipid accumulation, and the expression levels of selected mRNAs and miRNAs, as well as, AAK-2 protein abundance upon treatment with MACK (25, 50 and 100 μM), ORST (12 μM), or their hybrid combination (MACK 100 μM/ORST 12 μM) was performed. Collectively, this study validated in vivo the promising anti-obesogenic properties of MACK and provided mechanistic data on its further exploration as a potent plant-derived bioactive compound that benefits obesity management.

## 2. Results

### 2.1. Maackiain Acts as a Chemoattractant and Increases Locomotor Activity in C. elegans

**Figure 1:**
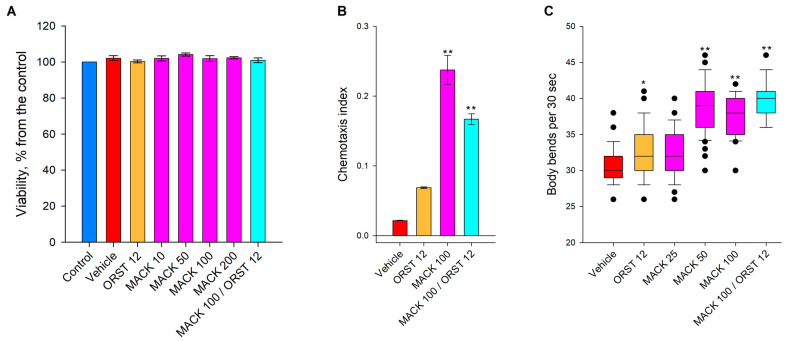
Maackiain (MACK), attracts glucose-supplemented *C. elegans* and elevates their motility. (**A**) Viability assessment to verify the safety of the selected concentrations of MACK, n = 12. (**B**) The effect of MACK (100 μM), orlistat (ORST, 12 μM) or their hybrid combination on the chemotaxis index (CI) compared to the vehicle, n = 15. (**C**) Bending movements of MACK (25, 50 and 100 μM) or ORST treated glucose-supplemented worms within 30 sec compared to the vehicle group, n = 45. Data are presented as mean ± SEM, * *p* < 0.05, ** *p* < 0.01. For comparison between the groups one-way ANOVA, followed by Tukey’s post hoc test were used.

We evaluated the potential cytotoxicity of MACK on human SGBS adipocytes in our previous study [14]. Similarly, within the current study, we provided data that MACK does not affect *C. elegans* viability up to concentrations of 200 µM (Figure 1A). Our data is consistent with the safety assessments performed in *C. elegans* [28], macrophages (RAW 264.7), mice, and *E. coli* [17] that were reported earlier. As the viability analysis revealed the absence of toxicity for MACK (Figure 1A), further chemotaxis and locomotion assays were performed.

The chemotaxis index was evaluated for assessing nematodes’ responses to supplementation with natural compounds. This index further serves as an indicator of the impact on neuroendocrine signaling, which regulates the feeding behavior of nematodes and is associated with body fat regulation [29,30,31,32]. During the chemotaxis assay, the worms showed preferences to both MACK 100 μM and its hybrid combination with ORST 12 μM, in comparison to the vehicle (Figure 1B), which additionally confirmed the lack of the toxicity of the treatments. An elevated bending rate is associated with the expenditure of energy [32,33,34]. Therefore, based on the observed increase in body bend upon the treatments (Figure 1C), we suggest that MACK, both alone and in combination with ORST, positively affects the energy expenditure.

### 2.2. Maackiain Reduces Lipid Accumulation in Glucose-Stimulated C. elegans

Nematodes store their triglyceride depots in small droplet-like organelles, termed as lipid droplets, which are primarily located in the intestine and hypodermis [18]. To quantify lipid accumulation, Nile red was employed to assess the effect of MACK, orlistat (as an approved anti-obesity medication), or their combination in glucose-fed worms. As previously reported, the supplementation with 2% glucose to the NGM serves as an obesogenic stimulus in *C. elegans* [18,26,27,33,34].

**Figure 2:**
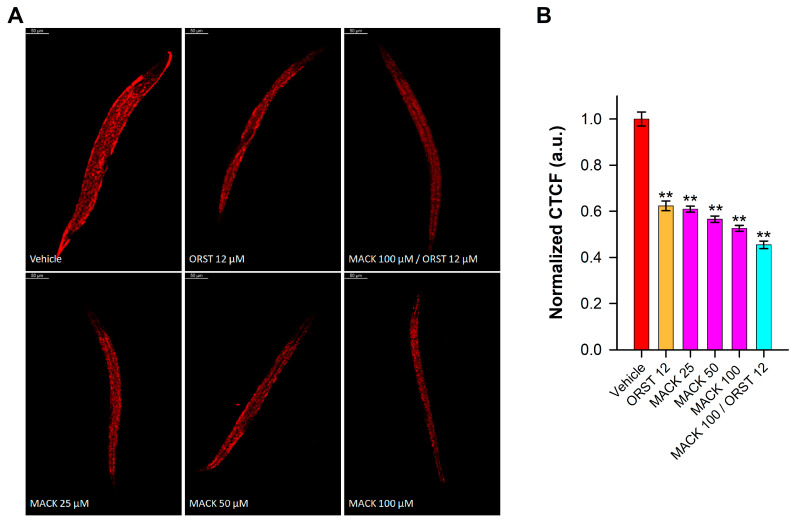
Maackiain (MACK) potentiated the lipid reducing activity of orlistat (ORST) in glucose-supplemented *C. elegans*. (**A**) Representative fluorescent confocal images (magnification 20×; scale bar 50 µm) of Nile red stained N2 nematodes, treated, respectively with MACK (25, 50 and 100 µM), ORST (12 µM), MACK/ORST (100/12 µM), or the vehicle. (**B**) Quantification of triglyceride accumulation, measured as the fluorescence intensity normalized to the vehicle group and presented as normalized corrected total cell fluorescence (CTCF), n = 90–100 worms per condition. Error bars indicate the mean ± SEM for normalized CTCF in arbitrary units (a.u.). Statistical significance between the groups was determined by one-way ANOVA, followed by Tukey’s post hoc test, ** *p* < 0.01 compared to the vehicle group.

The representative confocal microphotographs (Figure 2A) illustrated the tendency in the modulation of fat deposition in nematodes upon the different treatments. Nematodes treated with 25, 50, and 100 μM exhibited a dose-dependent and significant reduction in lipid accumulation, surpassing the effect of the lipid-reducing control, ORST (Figure 2B). Of particular interest was the superior decrease in lipid accumulation with the hybrid combination.

Our results affirmed the anti-obesogenic potential of MACK and its combination with ORST in glucose-fed *C. elegans*. Therefore, to investigate the molecular mechanisms involved in the effects of MACK, we have proceeded to gene expression analyses with its highest concentration and the hybrid combination.

### 2.3. Maackiain Upregulates Genes Associated with the Nutrient Sensing aak-2/sir-2.1 Signaling Pathway

Lipid metabolism in *C. elegans* encompasses multiple intricately regulated processes. In the current study, we investigated the expression profile of key transcription factors associated with the activation of the lipid biosynthesis, namely *sbp-1* and *cebp-2*. Moreover, as a functional homolog of human PPARs, we examined the *nhr-49* transcription factor which promotes two separate aspects of lipid metabolism—fatty acid desaturation and β-oxidation [22,25,26,27]. The relative mRNA expression of the *aak-2*, an ortholog of AMPK [35,36], as well as *sir-2.1* and *mdt-15*, was also assessed to determine whether the treatment with MACK affects these signaling pathways related to the nutrient and energy-sensing networks. The activation of these pathways is known to beneficially influence lipid metabolism and is hence associated with caloric restriction mimicking [12,21,25].

Maackiain treatment led to a significant and concentration-dependent decrease in triglyceride accumulation in glucose-supplemented nematodes. Therefore, analysis of the relative mRNA expression of nematodes treated with MACK (100 μM), ORST (12 μM), or the hybrid combination MACK/ORST (100/12 µM) shed light on the molecular mechanism involved in the observed anti-obesogenic effect.

**Figure 3:**
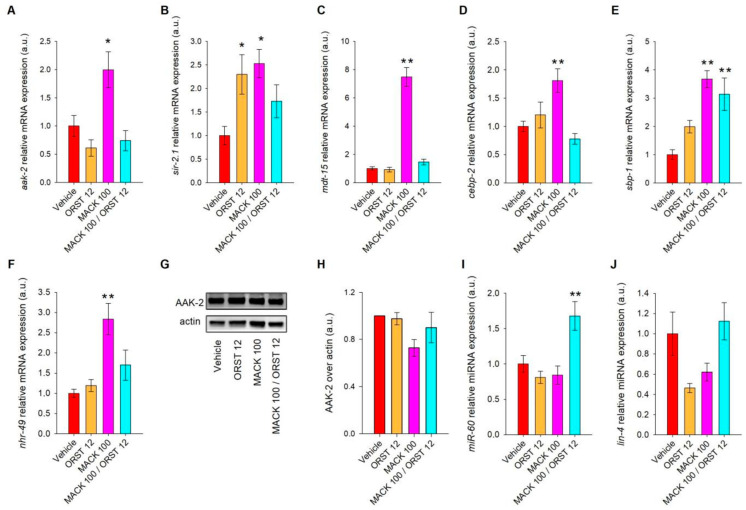
Maackiain (MACK) mimics caloric restriction in glucose-supplemented nematodes through upregulation of *nhr-49*, *aak-2*, *mdt-15*, and *sir-2.1*. Relative mRNA expression of genes involved in lipid metabolism upon treatment with MACK (100 μM), orlistat (ORST, 12 μM), or the hybrid combination MACK/ORST (100/12 µM), normalized to the vehicle and represented as arbitrary units (a.u.): (**A**) *aak-2*, (**B**) *sir-2.1*, (**C**) *mdt-15*, (**D**) *cebp-2*, (**E**) *sbp-1*, (**F**) *nhr-49*. The *mdh-1* and *iscu-1* were used as reference genes. (**G**) Phosphorylation levels of AAK-2 (pAMPK) in wild-type glucose-fed *C. elegans*, (**H**) normalized to actin as housekeeping protein. Relative miRNA expression profile of (**I**) *miR-60* and (**J**) *lin-4*, normalized to the vehicle expressed in a.u. Reference gene *U18* was used as was the exogenous control *ath-miR-159a*. Error bars indicate the mean ± SEM, where * *p* < 0.05, ** *p* < 0.01 compared to the vehicle. One-way ANOVA followed by Tukey’s post hoc test was applied for evaluation of statistical significance.

The single MACK treatment in the highest concentration applied (100 μM) significantly upregulated *aak-2*, *sir-2.1*, *mdt-15*, *nhr-49*, *cebp-2*, and *sbp-1* (Figure 3A–F) in comparison to the vehicle. Orlistat treatment triggered the significant upregulation of *sir-2.1* (Figure 3B), while its hybrid combination with MACK elevated the relative mRNA expression of *sbp-1* (Figure 3F).

The detected alternation in the relative mRNA expression of selected participants in the nematodes’ fat metabolism revealed that MACK activated the signaling pathways involved in fatty-acid oxidation, mitochondrial biogenesis, and energy expenditure, which are closely related to caloric restriction, namely *aak-2* and *sir-2.1*.

Activated AAK-2/AMPK in *C. elegans* has been associated with dietary restriction-dependent lifespan extension via remodeling mitochondrial function in peripheral tissues, increasing energy expenditure, and promoting healthy aging [20,21,26,35]. The observed transcriptional activation of *aak-2* upon MACK treatment has justified the need to detect the phosphorylation levels of AMPK at Thr172, a site in the activation loop associated with increased activity, in the glucose-induced *C. elegans* model (Figure 3G,H). The Western blot analysis at the selected timepoint did not reveal altered AAK-2 phosphorylation upon neither MACK nor the combination MACK/ORST. These results correspond to our previously reported data [14] on MACK anti-adipogenic activity in human adipocytes, as we detected AMPK overexpression and the lack of changes in protein phosphorylation AMPK levels.

Subsequent evaluation of the relative expression of miRNAs was employed in order to a obtain broader picture of the regulation of gene expression upon the experimental treatment in glucose-supplemented *C. elegans*. Recent studies have focused on exploring the potential of natural substances to modulate miRNAs associated with cancer, which also could be applied in obesity research [37,38,39,40,41]. Notably, several miRNAs in *C. elegans* are linked to lipid metabolism and ageing, including *mir-60* and *lin-4*. Furthermore, *mir-60* exhibits exclusive expression within the nematode’s intestines, exerting influence over their response to oxidative stress, nutrient absorption, and recovery from food deprivation [39,40,41]. The results of the RT-qPCR indicated that only the treatment with the hybrid combination MACK/ORST (100/12 µM) led to an upregulation of *miR-60* expression (Figure 3I). Interestingly, earlier studies established a connection between the downregulation of *miR-60* and lipid-reduction [41]. Nonetheless, our findings propose that increased expression of this microRNA might also play a role in lipid metabolism mechanisms. In our study, *lin-4* was not affected in all of the experimental treatments (Figure 3J). Thus, it is reasonable to suggest that the lipid-reducing effect of the hybrid combination is likely unrelated to *lin-4*.

### 2.4. Proposed Mechanism of the Anti-Obesogenic Effect of Maackiain in C. elegans

**Figure 4:**
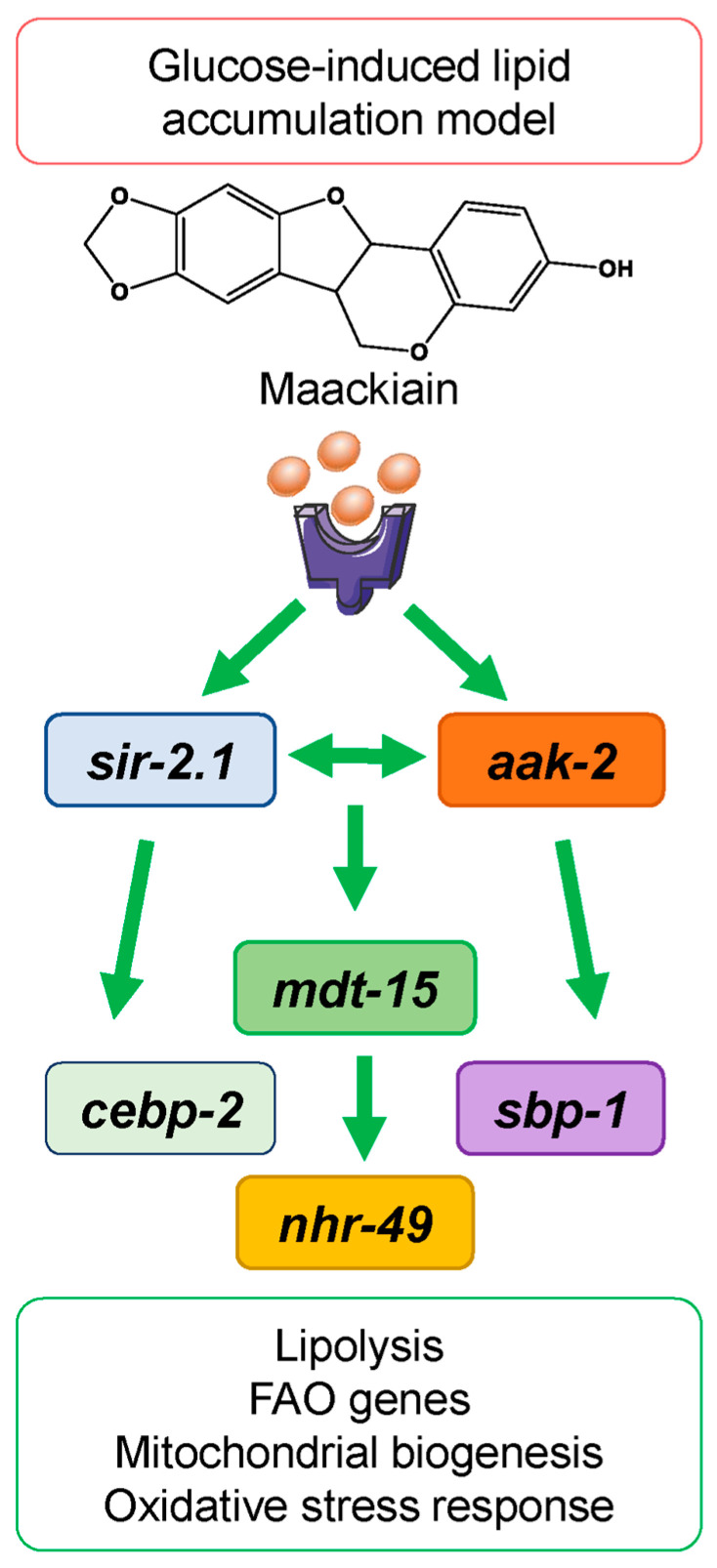
Maackiain reduces lipid accumulation by mimicking caloric restriction-induced *aak-2/AMPK* upregulation in *C. elegans*-based obesity model.

The present study provided mechanistic details on the anti-obesogenic activity of MACK that was suggested in our previous study on human adipocytes [14]. Here, we evaluated evolutionary conservation in the nematodes regulatory signaling pathways, corresponding to the lipid metabolism and energy expenditure in mammals. In this current investigation into the impact of MACK on the *C. elegans*-based obesity model, our findings elucidate the pronounced influence of this bioactive compound on lipid metabolism, reminiscent of the effects induced by certain caloric mimetic agents. Mechanistically, MACK upregulates pivotal sensory regulators within the nutrient and energy signaling network, including *aak-2*, *sirt-2.1*, *nhr-49*, *cebp-2*, and *sbp-1*, to a degree exceeding the effect of ORST and the MACK/ORST combination. Based on the results obtained from MACK treatment in glucose-induced lipid accumulation in *C. elegans*, the molecular mechanisms involved in anti-obesogenic activity are modeled in Figure 4.

In comparison to our prior exploration, treatment with MACK on a human adipocyte cell line exhibited distinctive results, characterized by the suppression of CEBPA and SREBP1, unaltered SIRT1 and PPARG expression, and an amplified expression of AMPK. In the context of the current experiment, where wild-type *C. elegans* were subjected to glucose-mediated lipid accumulation, we observed an opposing tendency of remarkable overexpression in the mRNA levels of *cebp-2* and *sbp-1*. Both the cell-based and the in vivo model systems consistently manifested a similar pattern of heightened AMPK/aak-2 expression. Notably, in the nematodes, the upregulation of *sir-2.1* is indicative of a potential collaborative interaction with *aak-2*, underscoring their contribution to the lipid-reducing efficacy of MACK in a manner that mimics caloric restriction. Inhibition in the transcription factor PPARγ, responsible for the differentiation of fat cells, has been identified as the major mechanism of action of MACK in human adipocytes. In contrast, the functional PPARs homologue *nhr-49* was positively regulated in *C. elegans* in the current experiment upon MACK treatment and is associated with *aak-2*/*sir-2.1*-dependent energy expenditure (Figure 4).

## 3. Discussion

Obesity is a multifactorial disease, commonly correlated with unbalanced dietary patterns and a lack of physical activity in individuals. Nonetheless, the risk factors contributing to augmented body mass encompass a deeper complexity, including genetic predisposition, prenatal metabolic reprogramming, epigenetic modifications, and endocrine disruptions [1,2,5,9]. Consequently, the management of obesity presents a significant challenge to the fields of both science and medicine [2,42,43]. In the contemporary landscape, obesity is surging even among the youngest of the population globally [44]. Therefore, comorbidities associated with increased weight, such as type 2 diabetes, have become a worldwide burden. Furthermore, obesity predisposes individuals to the development of cardiovascular and neurodegenerative diseases, as well as certain cancers [1,9]. These conditions further deteriorate the quality of life and pose significant challenges to the healthcare system. Thus, it is essential to develop more effective therapies for obesity [1,2,9,13].

Among contemporary strategies for weight reduction and the improvement of health, dietary regimens hold significant importance [5]. The timing of meal consumption has been thoroughly discussed and scientifically observed as a valuable approach for obesity prevalence [45]. Fasting, dietary restriction, and calorie restriction exemplify variants of weight-reduction strategies that have been proven to further mitigate inflammation associated with obesity, and to contribute to enhanced metabolism and overall well-being [5,12,45]. During calorie restriction, several vital metabolic sensors such as AMPK and SIRT1 are modulated to increase energy expenditure by mobilizing and oxidizing lipids [24,45,46]. However, for the majority of individuals diagnosed with obesity, altering dietary patterns alone may not be sufficient for significant weight reduction. Additionally, the challenges in adhering to these recommendations highlight the need for identifying substances that can trigger responses similar to caloric restriction in the body [12]. An innovative approach to obesity treatment, diverging from the conventional methods of dietary regimens, surgeries, and pharmacotherapy, explores the utilization of medicinal plants and their bioactive compounds [13,42]. Numerous phytochemicals have demonstrated their remarkable capacity to effectively regulate lipid metabolism, yielding favorable anti-obesity outcomes [18,47,48,49]. Furthermore, certain bioactive molecules, such as curcumin, exhibit characteristics that can influence the molecular networks associated with nutrient deprivation, effectively emulating the effects of dietary restriction [11,50,51]. Nevertheless, additional research is imperative to definitively validate the potential of plant-derived secondary metabolites in the management of obesity and its associated comorbidities.

As previously reported, MACK exerts a wide range of bioactivities, such as anti-allergic and anti-inflammatory [52], anti-cancer [53], and immunomodulatory against sepsis [17]. Moreover, MACK-containing plant extracts are beneficial for metabolic disorders such as diabetes [15,16]. Our previous findings in human adipocytes exposed that MACK has anti-adipogenic properties mediated by PPAR-γ-dependent inhibition of adipocyte differentiation and further modulation of key signaling pathways responsible for adipose tissue homeostasis [14]. In accordance with these results, the current study deals with the mechanistic evaluation of the anti-obesogenic effect of MACK, conducted in a glucose-induced obesity model in *C. elegans*. To our knowledge, MACK has been applied in *C. elegans* solely for the evaluation of its potential to alleviate Parkinson′s disease symptoms in a 6-hydroxydopamine-induced neurodegeneration model [28].

Complex molecular mechanisms are initiated in response to dietary restriction, playing a pivotal role as nutrient sensors critical for regulating metabolic processes, growth, and developmental pathways [12,45]. These intricate pathways exhibit a high degree of evolutionary conservation across diverse species. Despite the degree of genetic homology between humans and *C. elegans* being lower than that between humans and rodents, the nematodes represent a physiologically relevant model organism for studying lipid metabolism due to their completely sequenced genome [21,22,23]. For example, *C. elegans* is the recognized model for studying the role of AMPK and sirtuins in regulating cellular functions and their links to longevity and lifespan extension along with their role in lipid metabolism [20,33,54]. Similar to MACK, other bioactive molecules, such as caffeic acid, curcumin, and gallic acid, have demonstrated the capacity to mitigate fat accumulation through the modulation of caloric restriction [11,50,51]. The central component in their mechanism of action is AMPK/AAK-2, which has been similarly upregulated in the MACK treatment. Undetected phosphorylation of AMPK/AAK-2 in the current study could be due to different time point of the phosphorylation peak, which is reported for different activators [25,27]. Our prior data on human adipocytes also support the involvement of AMPK in the underlying mechanisms of MACK’s action [14], at least at the transcriptional level. Moreover, the correspondence observed between the results in locomotion and lipid staining implies an augmented energy expenditure, a factor frequently linked to enhanced autophagy, regulated nutrient signaling, and reduced oxidative stress [55]. These observations may also impact the neuroendocrine network, especially considering that MACK, both alone and in combination with ORST, serves as an attractant to the worms.

Recently, the approach of combining approved medicines with bioactive compounds of natural origin that aims to produce additive pharmacological effects, such as in the case of the dasatinib and quercetin senolytic combination that is under clinical evaluation for musculoskeletal disorders [56,57,58], has begun to be studied. Interestingly, our data on the hybrid combination between MACK/ORST showed superior effect in regard to lipid accumulation compared to application of each of the compounds alone. However, in regard to gene expression analyses, the combined treatment with MACK and ORST does not result in a synergistic interaction. A positive modulation was observed solely for *sbp-1* expression and the influence on the expression of *mir-60*. The absence of changes in *lin-4* expression upon treatment rather excludes an association between this microRNA and MACK’s lipid-reducing effect. Interestingly, the observed significant upregulation in the target genes upon MACK application alone is rather hampered when orlistat is added. Competitive interaction between the reference anti-obesity medicine ORST and the natural compound MACK that involves an overlapping molecular mechanism is one of the possible explanations of the observed reduction in the gene expression of the nematodes treated with the hybrid combination. Correspondingly, our findings suggest that both MACK and ORST upregulate *sir-2.1* expression while this effect is diminished upon their combination. Therefore, we could speculate that both compounds compete for their interaction within the sirtuin transcriptional regulation.

Our results substantiate that the administration of MACK, as well as its hybrid combination with ORST, extend the energy expenditure in the *C. elegans*-based obesity model and contributes to reduced lipid accumulation within both treatments. Detailed examination of the signaling pathways involved revealed distinct molecular responses. The treatment with MACK upregulated key transcription factors and co-activators integral to both oxidative lipid degradation, energy expenditure and mitochondrial activity (*aak-2*, *sir-2.1*, *mdt-15*, and *nhr-49*), and lipid biosynthesis (*cebp-2* and *sbp-1*). Conversely, the hybrid combination of MACK/ORST primarily heightened the expression of *sbp-1* and *miR-60*.

Taken together, the current study has validated the anti-obesogenic properties of the natural pterocarpan—maackiain, which has demonstrated superior efficacy in comparison to the applied control drug—orlistat. Additionally, the increased motility of the MACK-supplemented group was observed, indicative of elevated energy expenditure, concomitant with a reduction in the fat content in the nematodes. Mechanistically, the treatment with MACK activated molecular signaling pathways associated with caloric restriction, *aak-2/sir-2.1*, and energy expenditure and lipid metabolism, *nhr-49*/*mdt-15*. These findings provide a rationale for further investigation into the mechanisms underpinning the anti-obesogenic activity of MACK and its potential utilization as a natural compound with the capacity to mimic caloric restriction.

## 4. Materials and Methods

### 4.1. Materials

Maackiain (molecular weight 284.26 g/M; purity > 95% HPLC; Cat. № 83103) was supplied by Phytolab (Vestenbergsgreuth, Germany). Nematode growth medium (NGM; Cat. № MBS652667) was obtained from MyBiosource Inc. (San Diego, CA, USA). The LB broth Lennox (Cat. № L3022), agar powder (Cat. № 05039), M9 minimal salts (Cat. № M6030), fluoroshield histology mounting medium (Cat. № F6182), Nile red (Cat. № 72485), orlistat (Cat. № O4139), protease and phosphatase inhibitor cocktail (PPC1010), sodium hydroxide, and glucose were supplied by Sigma-Aldrich Co. (St. Louis, MO, USA). The reagents for RNA isolation, quantitative real-time polymerase chain reaction (RT-qPCR) analysis, as well as the consumables used for gel electrophoresis and immunoblotting analyses were supplied by Bio-Rad Laboratories Inc. (Hercules, CA, USA). Antibodies were used for Western blotting as follows: rabbit anti-phosphorylated AMPK (pAMPK, #2535) from Cell Signaling Technology (Leiden, The Netherlands); hFAB Rhodamine anti-actin beta (#12004164) and secondary StarBright Blue 700-conugated goat anti-rabbit IgG (#12004162) from Bio-Rad.

### 4.2. Caenorhabditis Elegans Maintenance and Treatment

The wild type N2 Bristol *C. elegans* and *Escherichia coli* OP50 were obtained from the Caenorhabditis Genetic Centre, University of Minnesota, Minneapolis, MN, USA, which is funded by NIH Office of Research Infrastructure Programs (P40 OD010440). The nematodes were grown at 20 °C according to standard procedures on NGM plates seeded with *E. coli* OP50 as a food source.

For the following experiments, a standard hypochlorite bleaching method of gravid adults was used to obtain an age-synchronized worm population [21,23,24,25,26,27]. Next, the nematodes were maintained on NGM plates supplemented with 2% glucose to model increased lipid accumulation [18,26,27]. During the L1 and L2 larval stage, *E. coli* OP50 was used as a food source. The supplementation of the experimental treatments was performed for the subsequent 24 h during the L3-L4 larval stages. The following substances—orlistat (12 μM), 0.4% DMSO (as a vehicle), MACK (25, 50, and 100 μM) or the hybrid combination between orlistat 12 μM and MACK 100 μM were added to heat-inactivated *E. coli* OP50. Then, the worms were transferred to fresh NGM plates with glucose seeded with the inactivated *E. coli* OP50 with or without treatments. Supplementing the nematodes with treatment compounds added to the heat-inactivated bacteria minimizes the possibility of biotransformation of the compounds by *E. coli* itself [28]. The safety of the concentrations for MACK used in the experiments was verified with a viability assay, performed as previously described [18]. Subsequently, L4 nematodes from each group were subjected to chemotaxis, and locomotion assays or collected for lipid staining, RNA isolation or Western blot, as described in the following subsections.

### 4.3. Locomotion Assay

The number of body bends exhibited by the nematodes following the experimental treatment was assessed through the previously described methodology by Savova et al. [18]. The treated nematodes were carefully transferred to a drop of M9 buffer on an NGM plate and allowed to acclimatize for 30 s. Subsequently, the number of bending motions during locomotion was monitored over a 30-s period. No fewer than 15 worms from each group were included, which was performed in triplicate.

### 4.4. Chemotaxis Assay

The analysis of chemosensory responses yielded valuable insights into the affinity of nematodes for specific substances, along with offering indications of the state of the neuronal network [20,31]. The analysis was performed according to the well-established protocol [32]. Briefly, a Petri dish was divided into four quadrants, which were designated as the test (“T”) or control (“C”) zones, respectively. In the center of the Petri dish, around 100-150 nematodes were placed. Subsequently, the Petri dish was incubated for 1 h at 20 °C, followed by 30 min at 4–6 °C to immobilize the nematodes, facilitating the scoring process. The chemotaxis index (CI) was calculated according to the formula: CI = (Quadrant test area 1 + Quadrant test area 2) − (Quadrant control area 1 + Quadrant control area 2)/Total number of nematodes [32]. The analysis was performed in three independent biological repeats.

### 4.5. Nile Red Triglyceride Staining and Confocal Imaging

To quantify triglyceride accumulation in the studied groups, Nile red lipid staining was employed, followed by the acquisition of fluorescent confocal microphotographs. Approximately 1500 nematodes (L4 stage) were treated as described in Section 4.2. The lipid staining and preparation of microscopic specimens were performed as described by Savova et al. [18]. The confocal system Stellaris 5 with the DMi8 inverted microscope from Leica (Wetzlar, Germany) was employed for capturing the confocal images. The fluorescence density value was calculated using ImageJ software version 1.53t and normalized to the vehicle group. The results were expressed as corrected total cell fluorescence (CTCF), as previously described by Stuhr et al. [34].

### 4.6. Gene Expression Analysis through RT-qPCR of mRNA and miRNAs

Approximately 3000–4000 nematodes per group, treated with the vehicle (to final concentration of 0.4% DMSO), MACK 100 μM, and the hybrid combination between orlistat 12 μM and MACK 100 μM, were collected for the total RNA isolation using PureZol (Bio-Rad) according to the protocol of the manufacturer. The integrity and quantity of the extracted RNA were assessed using agarose gel electrophoresis and UV spectroscopy. First strand cDNA synthesis kit (Canvax, Cordoba, Spain) was applied for the reverse transcription of mRNAs. The CFX Maestro 1.1 software version 4.1.2433.1219 (Bio-Rad) was used to quantify the expression of mRNAs using the ΔΔCT method. As endogenous control mRNAs, *iscu-1* and *mdh-1* were used, and the results were normalized to glucose-supplemented vehicle. The primers’ nucleotide sequences, which were used to analyze the relative mRNA expression, are listed in Supplementary Table S1.

Using stem-loop primers [37] and the Revert Aid H Minus First Strand cDNA Synthesis kit from Thermo Fisher Scientific (Waltham, MA, USA), the reverse transcription of miRNAs was carried out. The miRNA expression was measured using the ΔΔCT method, normalized to the vehicle using CFX Maestro software (Bio-Rad). Reference miRNAs included were endogenous *U18* [38] and the exogenous control *ath-miR-159a*. Supplementary Table S2 contains a list of the primers required for cDNA synthesis and qPCR.

### 4.7. Western Blot Analysis

Total protein lysates from the nematodes were prepared using ice-cold RIPA buffer (1% protease phosphatase inhibitors) and quantified by Bradford reagent [35]. Equal amounts of total protein lysates were subjected to 10% sodium dodecyl sulphate polyacrylamide gel electrophoresis, followed by transfer to nitrocellulose membrane, which was blocked for 1 h at room temperature in 5% (*w*/*v*) skimmed milk in Tris buffered saline. Overnight incubation with the primary anti-pAMPK antibody was performed, followed by 1 h with SB700-conjugated secondary antibody [21,26]. Multiplex fluorescent detection was visualized performed on a ChemiDoc MP imaging system (Bio-Rad). The abundance of the protein of interest was normalized against β-actin by means of Image Lab 6.0.1 software (Bio-Rad). An uncropped image of the blotting membrane is provided in Supplementary Figure S1.

### 4.8. Statistical Analysis

The obtained results were subjected to analysis in SigmaPlot v11.0 from Systat Software GmbH (Erkrath, Germany). Each assay is performed in three independent biological experiments and the data are represented as the mean ± SEM. The Shapiro–Wilk was used as a normality test to assess the data distribution. The statistical significance between groups was calculated by one-way analysis of variance (ANOVA), followed by Tukey’s post hoc test and is denoted as * *p* < 0.05, ** *p* < 0.01, compared to the vehicle, respectively. Confocal microphotographs of the lipid staining were representatively selected amongst the images form three independent experiments.

## Data Availability

All relevant data are within the manuscript. The data set generated and analyzed during the current study is also available from the corresponding author upon request.

## Supplementary Materials

The following supporting information can be downloaded at: https://www.mdpi.com/article/10.3390/ijms242417442/s1.
