# Supplementary material for: Maackiain Mimics Caloric Restriction through aak-2-Mediated Lipid Reduction in Caenorhabditis elegans
Source: Int J Mol Sci. 2023 Dec 13;24(24):17442. doi: 10.3390/ijms242417442 (PMC10744277; doi:10.3390/ijms242417442)
Supplement: Supplementary file 1 [file ijms-24-17442-s001.zip › ijms-2765459-supplementary.pdf]

## Supplementary information

# Maackiain is mimicking caloric restriction through *aak-2*-mediated lipid reduction in *Caenorhabditis elegans*

**Saveta G. Mladenova [1,#], Martina S. Savova [2,3,#], Monika N. Todorova [2,#], Milen I. Georgiev [2,3] and Liliya V. Mihaylova [2,3,*]**

[1] Independent researcher, 1000 Sofia, Bulgaria; savimladenova@abv.bg
[2] Laboratory of Metabolomics, Institute of Microbiology, Bulgarian Academy of Sciences, 139 Ruski Blvd., 4000 Plovdiv, Bulgaria; mntodorova@yahoo.com; m.sav@abv.bg; liliya.vl.mihaylova@gmail.com; milengeorgiev@gbg.bg
[3] Department of Plant Cell Biotechnology, Center of Plant Systems Biology and Biotechnology, 4000 Plovdiv, Bulgaria; m.sav@abv.bg; liliya.vl.mihaylova@gmail.com; milengeorgiev@gbg.bg
* Correspondence: liliya.vl.mihaylova@gmail.com; Tel.: 00359 32 64 24 30
# Shared first authorship

[*]**Corresponding author**: Liliya V. Mihaylova, Laboratory of Metabolomics, Department of Biotechnology, Institute of Microbiology, Bulgarian Academy of Sciences, 139 Ruski Blvd., 4000 Plovdiv, Bulgaria; e-mail: liliya.vl.mihaylova@gmail.com; tel: 00359 32 64 24 30.

## Supplementary Tables

**Table S1. Primer sequences, used in RT-qPCR of mRNAs**

| Gene | Forward | Reverse |
|---|---|---|
| *aak-2* | GGACGTCATTGCTCACGAGTGG | ATAGCGCTCAGTGACCTCTCGG |
| *cebp-2* | AGCGATGAGTGGAAATCGGAAGCG | GTCGGGTTCTGTTCACAGCTTCGT |
| *iscu-1* | CTCCTGCACAAGTTTGCGTTGC | CCGACGCTTGGATCGTTCTTGT |
| *mdh-1* | GGACCATTCATCGCCACTGTCC | TGTGATCACAAGCGGCCTTAGC |
| *mdt-15* | ATGGAAGCAGCCAGATGAATGG | CCGTAACCACCGTATCCATTCG |
| *nhr-49* | ATGCGCGTGTCGTTATTGTCGC | AGTCCCGTTGAGACTGGCTCCAG |
| *sbp-1* | AGCGTCGCATGCTCACTCTTCC | TACGAGCTCACGTGCCGAATGC |
| *sir-2.1* | TGTGTTTGTTTCGGGTGCATCGG | AGAAGTTGCGGTCACACACGGG |

**Table S2. Primer sequences, used in quantitative cDNA synthesis and RT-qPCR of microRNAs (miRNAs)**

| miRNA | Stem-loop primer | Forward | Universal reverse |
|---|---|---|---|
| ***cel-miR-60-3p*** | GTCGTATCCAGTGCAGGGTCC GAGGTATTCGCACTGGATACG ACTGAACT | AGCCAGCGTATT ATGCACATTTT | CCAGTGCAGGG TCCGAGGTA |
| ***cel-miR-lin-4-5p*** | GTCGTATCCAGTGCAGGGTCC GAGGTATTCGCACTGGATACG ACTCACAC | GACATCCCTGAG ACCTCAAG | |
| ***ath-miR159a*** | GTCGTATCCAGTGCAGGGTCC GAGGTATTCGCACTGGATACG ACTAGAGC | AAGCGCCTTTTG GATTGAAGG | |
| ***U18*** | GTCGTATCCAGTGCAGGGTCC GAGGTATTCGCACTGGATACG ACTGGCTC | AACAAGTGGCAG TGATGATCAC | |

## Supplementary Figures

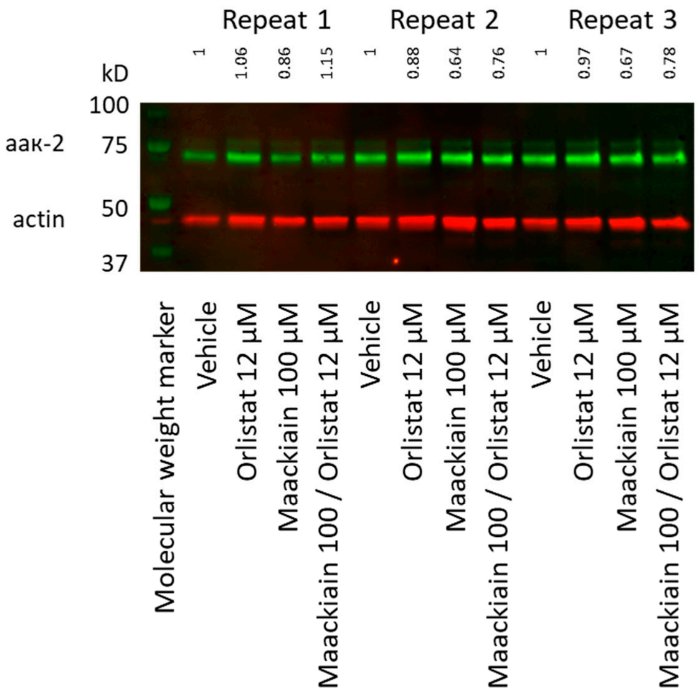


| MEAN | SEM | |
|---|---|---|
| 1 | | Vehicle |
| | | |
| 0.974908 | 0.052519 | Orlistat 12 µM |
| | | |
| 0.727871 | 0.071101 | Maackiain 100 µM |
| | | |
| 0.900613 | 0.128184 | Maackiain 100 / Orlistat 12 µM |

**Figure S1. Whole uncropped Western blots images from which the protein bands for Figure 3G.** The All Blue Standards (#1610373, Bio-Rad) was added to mark the positions of molecular weight. The loaded samples were vehicle, orlistat 12 µM; Maackiain 100 µM and the hybrid combination between maackiain 100 µM /orlistat 12 µM. For the determination of the protein's concentration, specific rabbit anti-pAMPK primary antibody (#2535, Cell Signaling Technology) and an anti-rabbit secondary fluorescent antibody StarBright 700 (#12004161, Bio-Rad) were applicated, the green bands. β-actin were used as loading control for Western Blot to normalize the levels of protein detected by confirming that protein loading is the same across the gel. The hFAB™ Rhodamine housekeeping protein fluorescent primary antibodies (#12004164, Bio-Rad) bands in red. Western blot analyses were conducted by ImageLab 6.0.1 software (Bio-Rad).
